# Comparisons between different methods of calculating dynamic strength index: Effect on training recommendations

**DOI:** 10.1371/journal.pone.0331519

**Published:** 2025-09-18

**Authors:** Nicholas Joel Ripley, Jack Fahey, Stuart Guppy, Paul Comfort

**Affiliations:** 1 School of Health and Society, University of Salford, Salford, United Kingdom; 2 School of Medical and Health Sciences, Edith Cowan University, Joondalup, Australia; Università degli Studi di Milano: Universita degli Studi di Milano, ITALY

## Abstract

An impulse-based dynamic strength index (iDSI) that constitutes time-dependent force expression in contrast to the common peak force-based DSI (fDSI) may be more insightful when considering training recommendations. However, a limitation of the iDSI is that any change in countermovement jump (CMJ) propulsion phase duration could affect longitudinal comparisons. A fixed duration (e.g., 250 ms) iDSI however could overcome this limitation. The purpose of the study was to determine the effect of different methods of calculating DSI (fDSI, iDSI matched, iDSI fixed) on the DSI value and resultant training prioritization. Thirty-seven team sport athletes (female = 13, male = 25, age = 22.2 ± 2.8 years, height = 174.4 ± 6.0 cm, mass = 74.9 ± 11.9 kg) performed three maximal effort CMJ and three isometric mid-thigh pull (IMTP) trials on force plates Fixed iDSI was identified from IMTP onset-250 ms, while matched iDSI was identified from IMTP onset-matched to CMJ propulsion phase duration. Participants were characterized using thresholds >0.80, 0.60–0.80 and <0.60 for maximal force, balanced, and dynamic force-based training, respectively. Mean ± SD of fDSI, fixed iDSI and matched iDSI were 0.82 ± 0.12, 0.88 ± 0.11 and 0.82 ± 0.11, respectively. Fixed iDSI was significantly and meaningfully greater than both fDSI and matched iDSI (*p* < 0.049, *d* > 0.408), with no differences between the latter (*p* = 1.000, *d = *0.007). There were large intra-individual differences in the training recommendations, 44.7% of the recommendations were consistent between fDSI and matched iDSI and 55.3% were consistent for fDSI and fixed iDSI. In contrast, there was a greater consistency in training recommendations between fixed iDSI and matched iDSI (84.2%). Despite the consideration of time-dependent force expression in iDSI calculations there are meaningful differences in their observations. This difference should be considered when tracking physical performance over time, where a fixed iDSI could be preferential to ensure consistency.

## Introduction

Lower limb strength is crucial for athletic performance [1], with meaningful associations with multiple measures of dynamic athletic performance (jumping, sprinting, changing direction and dynamic lift) [1]. Additionally, meaningful associations with sport performance have also been identified [1], with stronger athletes exhibiting improved technical skills such as tackle technique in sports such as rugby league [2]. Moreover, lower limb strength and the continued development of lower limb strength in team sport athletes is crucial in reducing the risk of lower limb injury incidence [3]. Greater lower limb strength has also been observed to decrease markers of muscle damage following competitive match play, improving recovery rates within team sport athletes [4]. This comprehensive picture highlights the necessity of lower limb strength and developing maximal lower limb strength. However, as highlighted bySuchomel, Nimphius (1), there is a point of diminishing returns, where further emphasis of lower limb strength development could be detrimental to overall athletic performance due to the time required to gain the marginal improvements in lower limb strength. This dedicated emphasis on maximum strength development may take away from the development of other key characteristics such ballistic and reactive force production.

One way that practitioners can easily group individuals into general training recommendations is to use the dynamic strength index (DSI), which is a ratio score of an athlete’s maximal dynamic capabilities and maximal isometric capabilities [5]. A DSI of <0.60 suggests good levels of maximal strength in comparison to their ability to express force during ballistic tasks, and that such athletes should focus on ballistic capabilities. In contrast, a DSI of 0.60–0.80 suggests balanced levels of strength and rapid-force expression capabilities during a dynamic action and that the athlete should have a combined training programe with an equal focus on ballistic and strength characteristics. Conversely, those athletes with a DSI > 0.80 have been suggested to poses an inadequate level of maximal strength compared to their expression of ballistic force, and therefore athlete should focus on maximal strength capabilities in order to improve overall athletic performance [5]. If a DSI score is greater than 1.00, this could be representative of either lack of understanding and familiarization within the isometric task or adopting an overly stiff strategy in the ballistic task, as isometric forces should be greater than dynamic force production [5]. This grouping enables practitioners to easily identify training needs, although practitioners should be aware of the limitations of this type of prescriptive guide as traditionally the DSI is based off peak force (PF) in the isometric task and peak propulsive force in the countermovement jump (CMJ) [6]. However, these are instantaneous values, which when considering the recommended sampling rate for force plates during dynamic tasks (1000 Hz) [7], equates to one sample per 0.001 s, and therefore may not provide a true reflection of an athlete’s force generating characteristics.

The most frequently used isometric and dynamic tasks to create the DSI are the isometric mid-thigh pull (IMTP) and the CMJ, although previous work has also used the isometric squat and squat jump [5]. However, Comfort et al. [6] reported improved within and between session reliability (intraclass correlation coefficient (ICC)) and decreased variability (coefficient of variability (CV)) when using the CMJ compared to the SJ. McMahon et al. [8] suggested key phases of the CMJ (unweighting, braking and propulsion), with propulsion peak force used for the calculation of the DSI. Interestingly, the shape of the force-time curve and the strategy of the jump can have implications on the outcome of the jump [9], therefore, incorporating the propulsive phase duration into the DSI could have implications on the outcome and recommendations of the DSI. James and Comfort [10] and Haischer et al. [11] have investigated incorporating different temporal-based DSI methods previously, with James and Comfort [10] identifying that utilizing force at specified time points within the DSI presented mixed within session reliability (acceptable absolute reliability, unacceptable relative reliability) but could offer additional insights into an athlete’s force generating capacity compared due to limited commonality with the conventional DSI. Haischer et al. [11] observed an impulse based DSI (iDSI), which incorporates force and time, with the authors matching the propulsive phase duration for the impulse calculation of the CMJ and IMTP. However, matching the impulse expression could lead to misinterpretations if an exceedingly long CMJ propulsive phase duration is observed or changes propulsive phase duration through application of training or injury. A fixed IMTP impulse duration however could offer an alternative assessment but is yet to be investigated.

Therefore, the primary aim of this study was to determine the effect of different methods of calculating DSI (force DSI [fDSI], iDSI matched, iDSI fixed) have on training prescription recommendations, including determining the relationship between DSI variations. As fDSI is typically the only method used within practice to give prescriptive recommendations, the lack of consideration for the time related factors of propulsive duration and incorporating impulse into the calculation could lead practitioners to miss key insights relating to an athlete’s force generating characteristics and lead to suboptimal programming decisions. The secondary aim of the present study was to determine the reliability of iDSI matched and iDSI fixed to determine if it could be used reliably within practice to inform training decisions. It was hypothesized that there would be differences in DSI values based on fDSI and iDSI, with no difference between iDSI variants with a meaningful relationship between the iDSI values. It was also hypothesized that all DSI variations would be reliable based on reliable collection of CMJ and IMTP trials. This may provide practitioners with a method of determining training needs, while still considering the time related factors which are crucial in most sporting actions. This could allow more optimised programming decisions to enhance athlete performance.

## Materials and methods

An observational cross sectional research design was used whereby dynamic and isometric force production characteristics of the lower limb was assessed on a single occasion, between 1^st^ July 2022 and 1^st^ December 2022. Upon arrival at the laboratory, written and informed consent was sought prior to testing and completing a participant readiness health questionnaire, subsequently had their height and mass recorded to the nearest 0.1 cm and 0.1 kg. Each subject then performed the normal dynamic warm-up that they would complete prior to their gym-based training, consisting of low intensity aerobic exercise and body weight exercises including squats, lunges, hip hinges followed by low intensity plyometric tasks. Ethical approval was granted by the School of Health and Society at the University of Salford (#1819−103), conforming to the Declaration of Helsinki (2013). Based on the work by Borg et al. [12], a-priori sample size estimation was performed using an expected ICC of >0.80, an alpha error probability *p < 0.05* and statistical power of 80% a required sample of 33 was identified. In order to observe if relationships exist between methods of calculating DSI, with an alpha error probability *p < 0.05,* statistical power of 80% and an *r* value of 0.41 (James and Comfort, 2022), a minimum sample of 33 was required. The sample size estimation was calculated using G*Power (Version 3.1, University of Deusseldorf, Germany).

### Participants

Thirty-seven team sport athletes (female = 13, male = 24, age = 22.6 ± 2.7 years, height = 175.4 ± 5.8 cm, mass = 74.2 ± 11.4 kg) participated within the present study. Based on the recommendations by McKay et al. [13], they would be categorized as tier 2–3.. Participants were from a variety of field-based team sports (Rugby League (6 male, 2 female), Rugby Union (6 male, 5 female), Soccer (11 male, 4 female), Field Hockey (1 male, 2 female)), volunteered to participate in this investigation. Phase of the menstrual cycle was not considered as this has been shown to have a trivial effect on force production characteristics [14–16].

### Procedures

Upon arrival at the laboratory, each subject provided written informed consent and subsequently had their height and mass recorded to the nearest 0.1 cm and 0.1 kg. Each participant then performed the normal dynamic warm-up that they would complete prior to their gym-based training, which included 3–5 minutes bike, squats, lunges, hops, submaximal jumps and submaximal mid-thigh pull with a 20 kg barbell. Prior to arrival all participants were advised to avoid food and caffeine 1 hour before and be in a well hydrated state.

### Countermovement jump

Three maximal-effort CMJ’s were performed with ground reaction forces recorded at 1,000 Hz using a previously zeroed force platform force platform (type 9286AA, Kistler Instruments, Winterthur, Switzerland) that was interfaced with a laptop computer and specialist software (Bioware 3.1, Kistler Instruments, Winterthur, Switzerland). The subjects were instructed to stand still for the initial one second of data collection [17,18], to enable the subsequent determination of their body weight (vertical force averaged over the first 1 second). The subjects were then instructed to perform the maximal-effort CMJs as fast and as high as possible, while keeping their arms akimbo [19]. Any jumps that were inadvertently performed with the inclusion of arm swing or leg tucking during the flight phase (tester observation) were omitted, and additional jumps were performed after 1 minute of rest. The mean value of the three trials was taken forward for analysis.

### Isometric mid-thigh pull

The IMTP assessments were conducted in line with previously recommended standardised procedures [20–22]. An immovable cold rolled steel bar was positioned at a height that replicated the start of the second pull phase of the clean in a custom rack (Absolute Performance, Cardiff, UK) while the athletes stood on a force platform (type 9286AA, Kistler Instruments, Winterthur, Switzerland) interfaced with a laptop computer and specialist software (Bioware 3.1, Kistler Instruments, Winterthur, Switzerland) and sampling at 1000 Hz. Once bar height was established, and the force plates zeroed, subjects then stood on the force platform with their hands strapped to the bar using standard lifting straps. Each subject adopted a posture which replicated the start of the second pull phase of the clean, resulting in knee and hip angles of 137.2 ± 4.1˚ and 145.9 ± 3.0˚ respectively, in line with previous recommendations [20–22].

After the dynamic warm-up, each subject performed three warm-up trials; one at 50%, one at 75% and one at 90% of their perceived maximum effort, separated by one minute of rest. Once body position was stabilized (verified by watching the subject and the force-time record), the subjects were given a countdown of “3, 2, 1, Pull”. Any obvious pre-tension, determined as a force >50 N above the subjects’ body mass, was not permitted prior to initiation of the pull, with feedback regarding this provided throughout the warm-up trials and the maximal effort trials. Subjects were instructed to “push their feet into the ground as fast and hard as possible”, in line with previous recommendations [20]. Each IMTP trial was performed for ~5 seconds, after at least one second of quiet standing in position prior to the start of the pull [20]. Subjects were provided with strong verbal encouragement during each trial. Each subject performed three maximal IMTP trials interspersed with two minutes of rest between trials. If PF during all trials did not fall within 250 N of the best trial, the trial was discounted and repeated after a further two minutes of rest, in line with previous recommendations [20]. All subjects performed three acceptable trials within a maximum of five maximal effort attempts and no further procedures were required, therefore the mean value of the three trials was taken forward for analysis.

### Data analysis

Raw unfiltered, force-time data for both the CMJ and IMTP were exported for subsequent analysis into bespoke Excel spreadsheets (Microsoft Corp, WA, USA). For the CMJ, center of mass velocity was determined by dividing force (minus body weight) by body mass and then integrating the product using the trapezoid rule [17]. Instantaneous center of mass displacement was determined by twice integrating force data at each time point [17]. Onset of movement was identified in line with current recommendations [18]. In brief, the first 1 second of vertical force was averaged, and the standard deviation (SD) was calculated. This SD was then multiplied by 5, and the first force value greater than this value was identified. Finally, the point 30 ms before this value was identified and marked the onset of movement as recommended by Owen et al. [18]. Take-off and touchdown were identified when the force fell below and exceeded 5 times the SD of the flight phase force, respectively [8,19,23]. The CMJ phases were identified using the terminology recommended by McMahon et al. [8]. Specifically, the unweighting phase was defined as occurring between the onset of movement and the instant of peak negative velocity, the braking phase was defined as occurring between the instants of peak negative velocity (plus one sample) and zero velocity, and the propulsion phase was deemed to have started when velocity exceeded 0.01 m·s^-1^ (this usually occurred one sample after zero velocity) and finished at take-off [8,19,23]. Propulsion peak force was defined as the maximum value attained during the propulsion phase. Propulsive duration was defined as the time between the end of the braking phase and take-off, while propulsion impulse was identified as the area under the curve within the propulsion phase determined via the trapezoid rule. Jump height was derived from vertical velocity at take-off [17].

**Fig 1:**
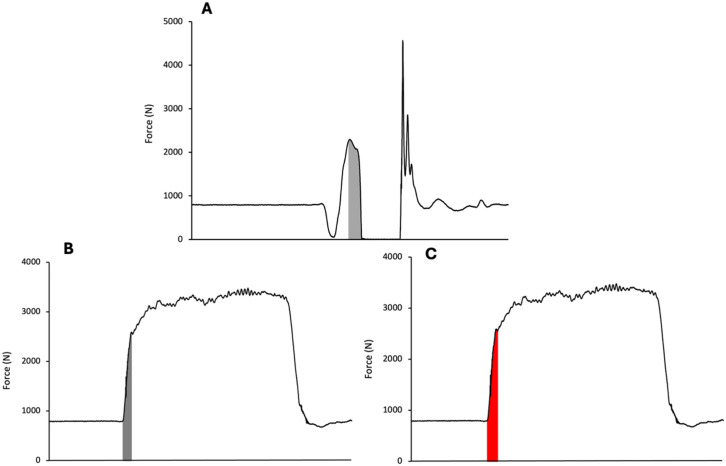
Visual representation of A) countermovement jump propulsive impulse (light grey), B) isometric mid-thigh pull matched impulse (dark grey) and C) isometric mid-thigh pull fixed 250 ms impulse.

For the IMTP, the onset of force production was defined as an increase in force that was greater than five standard deviations (SD) of the mean force calculated during the last 1 second immediately before the pull commence [20,24,25]. The PF was reported as the maximum force across the recorded absolute force-time curve. Impulse for the IMTP was identified as the area under the curve following onset as determined via the trapezoid rule. The duration of the IMTP impulse was matched to the impulse phase duration for iDSI matched and a fixed duration of 250 ms was used for iDSI fixed (Fig 1).

Conventional fDSI was calculated by dividing CMJ peak propulsive force by IMTP PF. iDSI fixed was calculated by dividing CMJ propulsive impulse by IMTP impulse from the onset of force production to 250 ms, while iDSI matched was identified by dividing CMJ propulsive impulse by IMTP impulse matched to CMJ propulsive phase duration. A 250 ms window was selected as it is a commonly threshold for fast stretch shortening cycle actions [26]. Interpretations on DSI were based on current recommendations [5], where values of <0.60, 0.60–0.80 and >0.80 would have training recommendations of ballistic training, combined ballistic and strength training and maximal strength training, respectively.

### Statistical analyses

Normality of all data was determined via the Shapiro-Wilk’s test, with all variables normally distributed (p > 0.05). Within session, between trial relative reliability was assessed using two-way mixed model ICC (model 3,1) and 95% confidence intervals (CI) [27]. The ICC values were interpreted based on the lower bound of the 95% CI as poor (<0.50), moderate (0.50–0.74), high (0.75–0.90) and excellent (>0.90) [27]. Within session, between trial absolute reliability was determined by calculating the percentage CV (%CV) and associated 95%CI, with <10% considered acceptable based off the upper bound of the 95%CI. The upper and lower bound 95% CI for the %CV was calculated by the addition or subtraction of 1.96 x standard error of %CV.

Pearson’s correlation coefficients were used to determine if any relationship existed between DSI variations, including bootstrapping with 10,000 replicate samples was used to increase the robustness of the analyses and enable reporting of the bias corrected 95% CI of the correlation between DSIs. Correlation coefficients were interpreted as trivial (0.00–0.09), small (0.10–0.29), moderate (0.30–0.49), large (0.50–0.69), very large (0.70–.089), nearly perfect (0.90–0.99) and perfect (1.0). Coefficient of determination was also calculated (R^2^).

A series of paired samples t-tests were performed to determine the difference between DSI variations, Cohen’s *d* effect sizes and associated 95% CI were also calculated and interpreted based on the recommendations of Hopkins [28] 0.00–0.19 = trivial and 0.20–- 0.59 = small, 0.60–1.19 = moderate and >1.20 = large. All statistical analysis was completed using JASP (JASP Team (2023). JASP (Version 0.18.1) [Computer software]).

## Results

All data were found to be normally distributed, with descriptive and reliability statistics presented in Table 1 and in the supplementary data file.

**Table 1:** Descriptive and reliability statistics for kinetic and ratio dynamic strength index scores.

	Mean ± SD	Range (min – max)	CV% (95% CI)	ICC (95% CI)
CMJ PPF (N)	1986.59 **±** 65.35	1421.82–2781.41	7.32 (5.45–9.19)	0.844 (0.813–0.865)
IMTP PF (N)	2499.21 **±** 75.10	1737.33–3837.01	6.01 (4.43–7.58)	0.885 (0.875–0.892)
CMJ PI (N s)	189.13 **±** 4.05	141.35–307.96	7.75 (5.78–9.72)	0.874 (0.860–0.884)
IMTP Matched impulse (N s)	234.55 **± **11.48	151.11–375.98	9.73 (5.76–11.81)	0.720 (0.665–0.853)
IMTP Fixed Impulse (N s)	216.14 **±** 9.21	150.08–314.99	9.92 (8.01–11.84)	0.733 (0.686–0.761)
fDSI (AU)	0.79 **±** 0.03	0.56–0.96	7.86 (5.86–9.84)	0.825 (0.785–0.853)
iDSI Matched (AU)	0.80 **±** 0.02	0.51–0.99	9.07 (6.83–11.32)	0.765 (0.746–0.778)
iDSI Fixed (AU)	0.87 **±** 0.02	0.48–0.98	8.54 (6.67–10.83)	0.755 (0.731–0.773)

CMJ = countermovement jump, PPF = peak propulsive force, IMTP = isometric mid-thigh pull, PF = peak force, PI = propulsive impulse, PD = propulsive duration, fDSI = force dynamic strength index, iDSI = impulse dynamic strength index, ICC = intraclass correlation coefficients, percentage coefficient of variation (%CV).

Matched iDSI and fixed iDSI demonstrated moderate relative reliability and unacceptable absolute reliability, whereas fDSI presented good relative reliability and acceptable absolute reliability.

**Fig 2:**
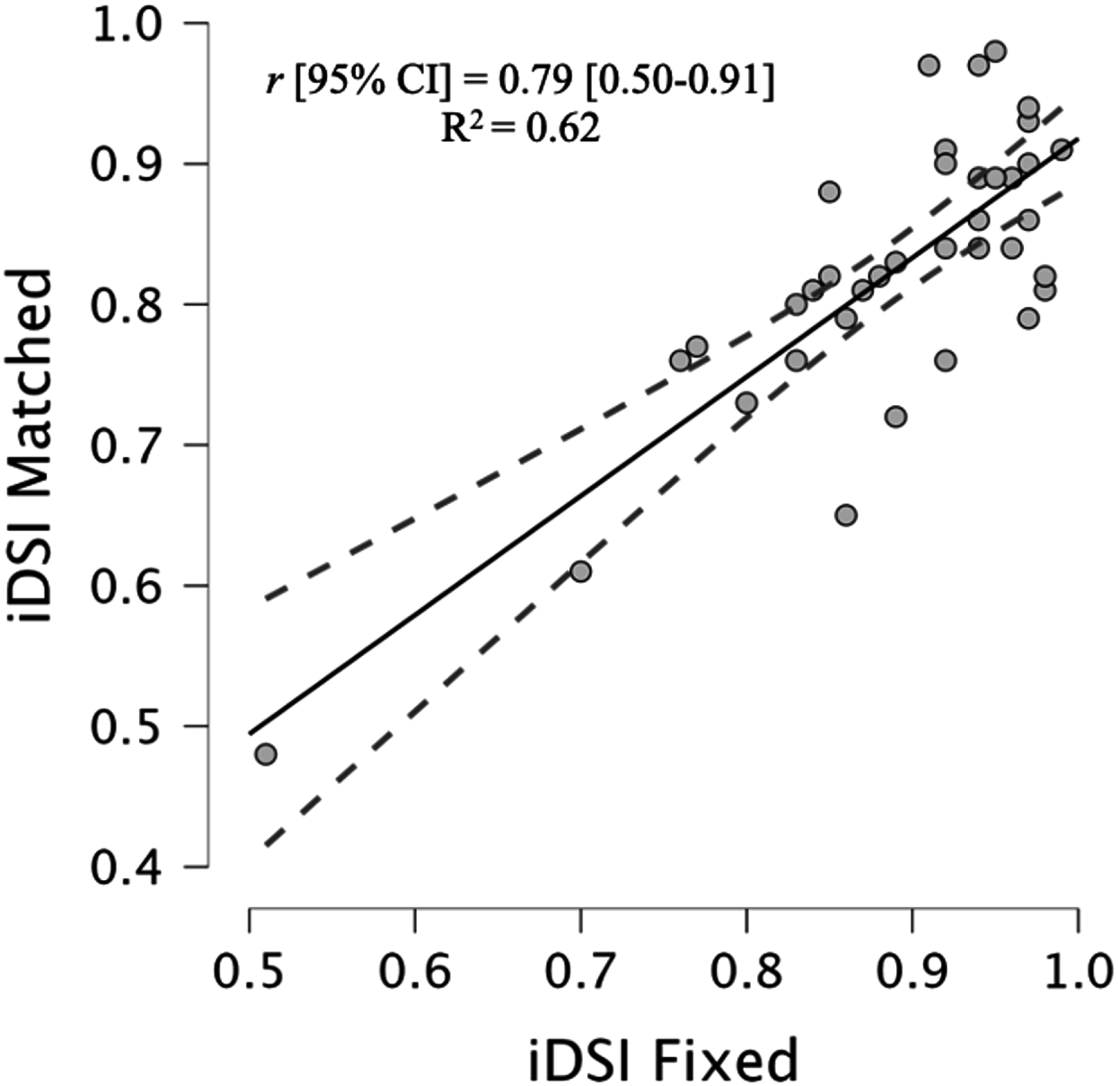
Correlation between iDSI matched and iDSI fixed and associated 95% confidence intervals.

Small-moderate associations were observed between fDSI and both variants of iDSI (iDSI Fixed: *r* [95% CI] = 0.29 [−0.22–0.56] & iDSI Matched: *r* 0.35 [−0.10–0.63]). A very large positive association was observed between iDSI fixed and matched (*r* [95% CI] = 0.79 [0.50–0.91], R^2 ^= 0.62) (Fig 2).

**Fig 3:**
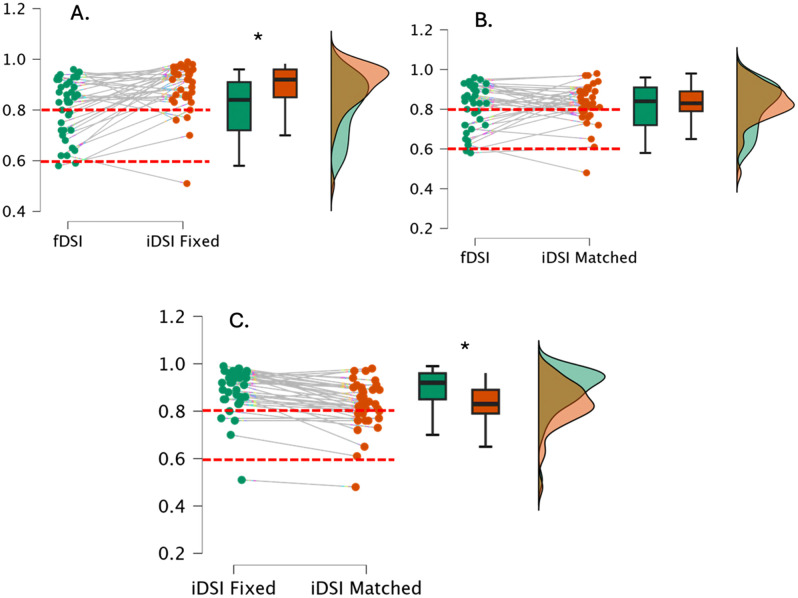
Scatter plots as a visual representation of pairwise differences between DSI variants with box and whisker plots and associated normal distribution (A. Force DSI compared to impulse DSI fixed, B. Force DSI compared to impulse DSI matched & C. Impulse DSI fixed compared to impulse DSI matched). Thresholds for training recommendations (ballistic <0.60 & maximal strength >0.80) are highlighted with dashed line. *denotes significant (*p < 0.001*) difference between DSI values.

Between DSI measures, fDSI and iDSI fixed had 56.8% (21/37) agreement on training recommendations with a moderate difference between DSI values (*p* < 0.001, *d (95% CI)* = 0.63 (0.27–0.98), fDSI and iDSI matched had 45.9% (17/37) agreement on training recommendations with a trivial difference between DSI values (*p* = 0.488, *d (95% CI)* = 0.12 (−0.21–0.44), while iDSI fixed and iDSI matched had 86.5% (32/37) agreement on training recommendations with a moderate difference between DSI values (*p* < 0.001, *d (95% CI)* = 1.03 (0.63–1.45) (Fig 3).

## Discussion

The primary aim of this study was to determine the effect of different methods of calculating DSI (fDSI, iDSI matched, iDSI fixed) have on the associated training prescription recommendations, while also determining the within session reliability of the DSI variants. The results of this demonstrate acceptable absolute reliability with good relative reliability for fDSI, whereas iDSI variants presented unacceptable absolute reliability and moderate relative reliability. There were also identifiable differences between DSI variants, suggesting that each variant potentially assesses different aspects of an athlete’s force generating capacity (peak and rapid force production). Trivial associations were found between fDSI and both iDSI variants, with between 45.9–56.8% agreement between training recommendations between fDSI and iDSI matched and fDSI and iDSI fixed, respectively.

The DSI provides practitioners with a guideline of training priorities, < 0.60 focus on ballistic capabilities, a DSI of 0.60–0.80 a combined training programe with an equal focus on ballistic and strength characteristics, a DSI > 0.80 focus on maximal strength capabilities [5]. This heuristic approach is particularly useful for practitioners, such as dual sport science and strength and conditioning coaches and those who are working with larger squads (such as team sports), where being time efficient is an essential skill. To apply this heuristic approach to profiling however, acceptable reliability is essential. Within the present study, fDSI and iDSI presented similar good levels of relative reliability, highlighting that they could be used to profile and rank order players. However, tracking acute changes could impacted by unacceptable absolute reliability (>10% CV based on the upper bound 95% CI). This contrasts previous literature, where excellent within session relative reliability (ICC = 0.970 (95% CI = 0.940–0.986)) and very good absolute reliability (CV = 4.69%) has been reported [5]. Although the authors did not report the 95% CI for absolute reliability [5], it is likely that this would have similar to those reported within the present study due to similar populations included within both studies. The increased variability within both iDSI variants could be related to the increased variability in CMJ time to take off and/or propulsive phase duration or variability within the performance of the IMTP such as the athlete performing either a slower ramped effort or a quicker impulsive effort which can be seen within the IMTP impulse variability. Between the three DSI methods, there was limited agreement in the training recommendations. When comparing fDSI and iDSI matched and iDSI fixed, recommendations agreed 44.7% and 55.3% of the time, respectively. This presents a question around the suitability of the training guidelines, especially with fDSI only considering a single time point where peak force is occurring in the CMJ or IMTP, whereas iDSI would consider the strategy and application of force.

Currently within the literature, only fDSI has been observed within training interventions [5,29]. Comfort et al. [5] observed changes in fDSI in collegiate athletes using a four-week low volume (2–3 sets of 3 repetitions) in-season strength training program (twice per week, weightlifting derivatives, back squat, Romanian deadlift and Nordic hamstring exercises, between 80–90% one repetition maximum). Excellent levels of reliability were observed by Comfort et al. [5], using the lower bound ICC 95% CI. Furthermore, the authors identified no significant or meaningful change in CMJ peak force, with a significant and small increase in IMTP peak force resulting in a significant and small decrease in fDSI [5]. Comfort et al. [5] performed sub-group analysis by dividing the group into high and low fDSI, with average fDSI values of 0.85 ± 0.05 and 0.56 ± 0.05, respectively. Following the completion of the strength training programe a non-significant and non-meaningful change in fDSI was observed in the low group, whereas the high fDSI group experienced a significant and large decrease, highlighting that those who were categorized as requiring a strength focus (DSI > 0.80) improved following a strength programe and supporting the potential use of the DSI thresholds. More recently, Pleša et al. [29] performed either strength or ballistic focused resistance training in highly trained basketball athletes based on fDSI, however they only observed a trivial decrease in fDSI. However, this is likely due to the differentiation of athlete requirements, where those ≤0.89 undertaking the ballistic focused program and ≥0.90 undertaking the strength focused program. This contrasts with previous recommendations, where all included athletes likely required a strength focused programe to see meaningful improvements [5]. Interestingly, the control group increased fDSI based off including a combination of both the strength and ballistic exercises further supporting the need for strength-based factors. However, as both the iDSI has not been observed following a training programe it may not provide similar response and requires further investigation. As iDSI also accounts for an athlete’s strategy of force application during the CMJ (i.e., propulsive phase duration vis propulsive phase impulse) it may offer further insights into an individual’s ballistic capabilities.

When compared to measures of physical performance, fDSI has significant, yet only small-moderate relationships between CMJ jump height, reactive strength index modified and IMTP rate of force development, whereas, unsurprisingly, IMTP peak force possessed a very large relationship with fDSI in both male and female athletes division 1 collegiate athletes [30]. As fDSI only accounts for one sample per 0.001 s during the CMJ, this could explain why there is minimal association with typical CMJ measures used by practitioners (i.e., jump height and reactive strength index modified), as it is only very small portion of CMJ performance and does not account for changes in movement strategy. However, if an iDSI was used then it might be expected to have a stronger association with these performance measures as it takes into account propulsive phase duration, while net propulsive impulse would dictate acceleration of mass and thus, take-off velocity and jump height [8,31]. McMahon and colleagues [32] observed CMJ performance characteristics of collegiate team sport athletes with either low and high fDSI value, with average fDSI values of 0.55 ± 0.10 and 0.92 ± 0.11, respectively. The low group fDSI group demonstrated significantly greater center of mass displacement, power, velocity and impulse in the braking phase, with greater phase time, center of mass displacement, velocity and impulse in the propulsive phase of the CMJ, these differences enabled the low DSI group to achieve a moderately greater jump height (although this was non-significant) [32], with likely differences only identified within the velocity and displacement characteristics across the CMJ waveforms. These results conform with the training recommendations for DSI [5], with high fDSI producing greater peak propulsive force with a stiff strategy and a lower center of mass displacement, hence requiring the ability to adopt a more compliant strategy to allow for greater propulsive phase duration and the ability to increase net propulsive impulse. Contrastingly, the low fDSI group adopted a compliant strategy with increased center of mass displacement, lower peak propulsive force requiring the ability to adopt a stiff strategy to enable increased peak propulsive forces. However, possible issues with this stem from the small relative observation period for fDSI (one sample per 0.001 s), whereas iDSI can account for jump strategy (propulsive phase duration and propulsive impulse) potentially having greater ability to examine differences in jump ability rather than jump strategy alone, although further investigation is required. Although force generating strategy cannot specifically be accounted for within the IMTP, an athletes ability to produce relatively high forces quickly within the IMTP could be impacting on iDSI [33,34]. Even though standardised cueing was used through the testing, the use of a 1 s short IMTP, where rapid force production is the aim [35,36], could provide iDSI with greater utility however this requires further investigation.

The present study is not without limitations. Despite familiarity with weightlifting derivatives and performing warm-up repetitions to familiarize subjects with appropriate cueing and performance, improved familiarity of IMTP performance could alter the findings of the present study. Further, a limitation of ratio-based metrics [37], such as the DSI, is that the practitioner needs to consider the constituent parts with an fDSI. In this scenario, the ability to interpret peak force (either absolute or relative) is more simplistic than an iDSI variation. Hence, the iDSI could be making interpretation of an athlete isometric and ballistic force generating capabilities more complex for practitioners. Similarly, observing the absolute performance of the dynamic task, such as the CMJ, would also be imperative with both fDSI and iDSI variations, as jump height, time to take off and propulsive phase duration highlight an athlete’s wider performance characteristics. However, the relationship to performance and the suitability of the training recommendations could be explored further within training interventions. Finally, it is worth noting that not all athletes would benefit from aiming for a DSI value between 0.60–0.80, which is the overall aim of prescribing exercise based on their respective score DSI and should be applied with caution. As specific positions in sports such as handball and basketball may benefit from specific strength profiles [38,39]. However, as these suggestions have only been made using fDSI, considering iDSI could present contrasting results.

## Conclusions

The DSI is a commonly used heuristic within strength and conditioning to enable quick decisions to be made on resistance training. However, the conventional fDSI may not provide a true reflection of the athletes’ force generating capabilities, accounting for a single peak value within a dynamic task. Hence, an iDSI could provide further context surrounding an athletes dynamic and isometric force generating capabilities, specifically as it would account for the propulsive duration within the CMJ. The results of the present study highlight that both iDSI match and iDSI fixed can be collected and assessed reliably, demonstrating an ability to profile athletes with good relative reliability. However, the DSI variants do present diverging training recommendations and hence could be identifying different performance characteristics, with differences related to jump strategy. It could be proposed that iDSI fixed could be more sensitive and useful for tracking longitudinal changes as the fixed duration IMTP impulse allows provides context on rapid and peak isometric force generating capabilities, while changes in dynamic propulsive duration (hence propulsive impulse) will alter the DSI value to determine future training needs. Whereas iDSI matched could result in similar DSI values even following training as the ratio will compare force generating characteristics over identical durations, hence the need to observe the constituent parts of the ratio metric. However, currently both iDSI fixed and iDSI matched need to further investigation to determine if the same absolute thresholds for determining training recommendations can be used for iDSI. Moreover, future research should also be conducted to determine the efficacy of training guidelines when using iDSI to determine if the greater context provided to an athlete’s dynamic force generating capability results in greater training effectiveness.

## Supporting information

S1 File Associated data can be found in the supplementary information file. (XLSX)
