# Supplementary material for: Comparisons between different methods of calculating dynamic strength index: Effect on training recommendations
Source: PLoS One. 2025 Sep 18;20(9):e0331519. doi: 10.1371/journal.pone.0331519 (PMC12445465; doi:10.1371/journal.pone.0331519)
Supplement: Attachment — Submitted filename: Response to reviewers.docx [file pone.0331519.s002.docx]

We thank both reviewer for their time and effort in reviewing our manuscript, we think it is now stronger with greater clarity in reporting and scientific rigour.

Please see replies and to all questions and comments in red below.

Reviewer #1: The followings are my questions to the authors:

1. Could you clarify the abbreviation "PPD" used in the abstract?

Thank you for noting this oversight, we have actually removed the abbreviation as it was only used once.

2. Why was a 250 ms window chosen for analysis? What are the implications if a subject reaches peak force significantly earlier than 250 ms? Were such cases observed in the study?

A 250 ms window was selected as a threshold as this is the commonly recognised threshold for fast stretch shortening cycle actions (i.e. plyometric actions). We have clarified this within the text with supporting reference.

Although this is an interesting point, it is highly unlikely that peak force will occur < 250 ms. No participants within the present study displayed this characteristic, this is consistent with previous literature in the isometric midthigh pull (Comfort et al., 2019) and in single joint actions (Andersen & Aagaard, 2006).

Comfort, P., Dos’Santos, T., Jones, P. A., McMahon, J. J., Suchomel, T. J., Bazyler, C., & Stone, M. H. (2020). Normalization of Early Isometric Force Production as a Percentage of Peak Force During Multijoint Isometric Assessment. *International Journal of Sports Physiology and Performance*, *15*(4), 478-482.

Andersen LL, Aagaard P (2006) Influence of maximal muscle strength and intrinsic muscle contractile properties on contractile rate of force development. Eur J Appl Physiol 96:46–52

3. Would a graphical representation of the three methods, incorporating CMJ and IMTP force-time curves, enhance the understanding of the methodologies?

Although this could be an interesting addition, we do not think it is necceasry as it has been previously illustrated effectively in the the work from James and Comfort (2022)

James LP, Comfort P. The reliability of novel, temporal-based dynamic strength index metrics. Sports Biomechanics. 2022;4(Feb):1-12.

4. Why was a 5SD threshold selected for force onset detection instead of manual detection? What are the advantages or limitations of this approach in particular to this new method in this study?

This threshold was used as it is recommended by Owen et al. (2014), which is a standard protocol for CMJ assessment.

5. As noted by Bishop (2024), ratio data can present certain limitations. Does the newly proposed method address these issues more effectively than existing methods?

Despite issues with ratio data it can still be useful and it is recommended that practitioners consider the constituent parts of DSI, and we acknowledge the potential that iDSI might be making it more complex (lines 423-430). We have included the Bishop reference to support this point.

“Further, a limitation of ratio-based metrics, such as the DSI, is that the practitioner needs to consider the constituent parts with an fDSI. In this scenario, the ability to interpret peak force (either absolute or relative) is more simplistic than an iDSI variation. Hence, the iDSI could be making interpretation of an athlete isometric and ballistic force generating capabilities more complex for practitioners. Similarly, observing the absolute performance of the dynamic task, such as the CMJ, would also be imperative with both fDSI and iDSI variations, as jump height, time to take off and propulsive phase duration highlight an athlete’s wider performance characteristics.”

6. In the tables, including range statistics could provide additional insight. Could you explain the implications of obtaining a DSI value greater than 1?

We have now included the range statistics. A comment around the achieving a DSI >1 has now been included within the introduction lines 91-93.

“If a DSI score is greater than 1.00, this could be representative of either lack of understanding and familiarization within the isometric task or adopting an overly stiff strategy in the ballistic task, as isometric forces should be greater than dynamic force production [5].”

7. The presented supplemental data appear to focus solely on ratios. Would it be beneficial to also include the raw data used to calculate these ratios?

We have now included the data as well.

8. Do the previously established 0.6 and 0.8 thresholds directly correspond to the new method, or have they been adjusted?

They have not been adjusted as there is no current recommendations to adjust, even when using different protocols. These are the recommendations from Comfort et al. (2018)

9. DOI number should be completed in references number 5 in the references list.

This has now been updated.

Reviewer #2: It seems that study design, evaluations and analysis were conducted in sound and profound fashion. The detail level about the conducted tests and measurements seems sufficient.

In order to increase the quality of the manuscript, you can find my recommendations below:

The Methods section should include information on the pre-test warm-up and nutrition, as both can have a substantial effect on the results.

We have provided some brief information on nutrition, however, as this is performed within-session (1 session) and not between nutrition would have a minimal effect on performance. We have provided detail on the warm.

The objective sentence (at the end of the introduction section) must be strengthened and must give more context about the importance and possible beneficial effects of the study

A practical beneficial statement has been added to this section.

At the end of the conclusions section, please provide point to point recommendations to sport scientists about how to make further adjustments to properly improve the quality of potential future research on this (or similar) topic.

Future research has been identified at the end of the conclusion. With appropriate suggestions on further adjustments and requirements to observe the constituent parts. Lines 458-466.

“Whereas iDSI matched could result in similar DSI values even following training as the ratio will compare force generating characteristics over identical durations, hence the need to observe the constituent parts of the ratio metric. However, currently both iDSI fixed and iDSI matched need to further investigation to determine if the same absolute thresholds for determining training recommendations can be used for iDSI. Moreover, future research should also be conducted to determine the efficacy of training guidelines when using iDSI to determine if the greater context provided to an athlete’s dynamic force generating capability results in greater training effectiveness.”

Best wishes
