# Supplementary material for: Comparisons between different methods of calculating dynamic strength index: Effect on training recommendations
Source: PLoS One. 2025 Sep 18;20(9):e0331519. doi: 10.1371/journal.pone.0331519 (PMC12445465; doi:10.1371/journal.pone.0331519)
Supplement: Attachment — Submitted filename: Response to editor and reviewer.docx [file pone.0331519.s003.docx]

Response to editor and reviewer

We thank both reviewers for their review and note that they both accepted the manuscript in its current form on the previous submission. We believe the manuscript is better for it.

We have now included the graphics as suggested by the editor and hope this is sufficient.
