# Supplementary material for: Comparisons between different methods of calculating dynamic strength index: Effect on training recommendations
Source: PLoS One. 2025 Sep 18;20(9):e0331519. doi: 10.1371/journal.pone.0331519 (PMC12445465; doi:10.1371/journal.pone.0331519)
Supplement: Attachment — Submitted filename: Response R3.docx [file pone.0331519.s004.docx]

Dear reviewer,

We have provided a detailed response to the feedback included below, obviously we would have done this in the first review if we had received all these comments.

We thank the reviewer for the discourse and comments highlighted.

REVIEWER 1:

Unfortunately, I could not thoroughly review the second revision of the manuscript because the authors did not receive my comments.

I provided the comments below (at the end) on the first revision of the manuscript. As I selected "Accept," I included my comments in the "Confidential to Editor" section, intending for them to be filtered by the Editor.

In the decision letter, the Editor referenced my comments, stating, “Please address the points made by the reviewers. I agree with the reviewers' request for graphics. I recommend that you make any necessary revisions to ensure that readers can best understand the article.”

However, it appears that the authors did not receive my comments. Consequently, the authors did not include a new graph in the second version of the manuscript.

Although we acknowledge this oversight and it unfortunate that we did not receive this on the first review, we do want to make the reviewer aware that the handling editor has changed multiple times since submission and to this point (three different editors).

My comments to the first revision of the manuscript:

I raised this point for the first version: “Would a graphical representation of the three methods, incorporating CMJ and IMTP force-time curves, enhance the understanding of the methodologies?”

The authors' response was: “Although this could be an interesting addition, we do not think it is necceasry as it has been previously illustrated effectively in the the work from James and Comfort (2022) James LP, Comfort P. The reliability of novel, temporal-based dynamic strength index metrics. Sports Biomechanics. 2022;4(Feb):1-12”

I still think a graphical representation would be useful. Unlike PLOS One, the article in which it is mentioned is not open source and is therefore not available to anyone. I understand that creating such a graph would be time-consuming, and the authors would prefer to publish sooner rather than later. In the long term, though, a graph would benefit authors and readers. Ultimately, the editors can make the final decision.

Although we do agree with our previous response around it being previously published figures. However, as the reviewer does feel strongly around this, we have created out figures which replicate those published previously. We do want to highlight that although the article is not open access, it is available on request from the authors, and it is not the responsibility of authors of newer works to replicate work that is behind a “pay wall” from other publishers.

I raised another point for the first version: “Why was a 5SD threshold selected for force onset detection instead of manual detection? What are the advantages or limitations of this approach in particular to this new method in this study?”

The authors' response was: “This threshold was used as it is recommended by Owen et al. (2014), which is a standard protocol for CMJ assessment.” 

I did not refer to the CMJ analysis. I referred to the IMTP analysis (For the IMTP, the onset of force production was defined as an increase in force that was greater than five standard deviations (SD) of the mean force calculated during the last 1 second
immediately before the pull commence [20]). Using 5 SD is not as standard as it is IMTP, unlike CMJ. Due to fluctuations in force caused by pre-tension and other factors, manual approaches are also common for force onset detection in IMTP analysis.

We apologies for the confusion based on the response, but as we used thresholds for both the CMJ and IMTP and your comment did not specify if it was the CMJ or IMTP we made a best guess and we overlooked the threshold for the IMTP when responding or we would have made this clearer.

Although the reviewer highlights some of the issues with a calculated onset threshold, you incorrectly suggest there no standard protocols for this, when in fact identifying onset through 5SD are the standardized methods recommended by Comfort et al. (2019). A threshold of 5 SD has also been supported in the literature by the work of Dos’Santos et al. (2017) over manual identification. Interestingly, Guppy et al. (2018) found both 5SD and 3SD agree with manual identification for time at force onset, while automated threshold also had improved reliability in force at longer epochs (i.e., >200 ms). Therefore, 5SD was used for the present study. We have now included both the Dos’Santos et al. (2017) and Guppy et al. (2018) references to support theuse of the 5SD onset threshold.

Comfort P, Dos’Santos T, Beckham GK, Stone M, Guppy S, Haff G. Standardization and Methodological Considerations for the Isometric Midthigh Pull. Strength and Conditioning Journal. 2019;41(2):1.

DosʼSantos T, Jones PA, Comfort P, Thomas C. Effect of Different Onset Thresholds on Isometric Midthigh Pull Force-Time Variables. J Strength Cond Res. 2017 Dec;31(12):3463-3473. doi: 10.1519/JSC.0000000000001765.

Guppy, S. N., Brady, C. J., Kotani, Y., Connolly, S., Comfort, P., Lake, J. P., & Haff, G. G. (2024). A comparison of manual and automatic force-onset identification methodologies and their effect on force-time characteristics in the isometric midthigh pull. Sports Biomechanics, 23(10), 1663-1680. <https://doi.org/10.1080/14763141.2021.1974532>
