# Supplementary material for: Comparisons between different methods of calculating dynamic strength index: Effect on training recommendations
Source: PLoS One. 2025 Sep 18;20(9):e0331519. doi: 10.1371/journal.pone.0331519 (PMC12445465; doi:10.1371/journal.pone.0331519)
Supplement: Peer review history

# Peer review: Decision Letter 0

**DOI:** 10.1371/journal.pone.0331519.r001

**Authors:** Zeki Akyildiz

9 May 2025

PONE-D-25-07136Comparisons between different methods of calculating dynamic strength index: Effect on training recommendationsPLOS ONE

Dear Dr. Ripley,

Thank you for submitting your manuscript to PLOS ONE. After careful consideration, we feel that it has merit but does not fully meet PLOS ONE’s publication criteria as it currently stands. Therefore, we invite you to submit a revised version of the manuscript that addresses the points raised during the review process.

Please submit your revised manuscript by Jun 23 2025 11:59PM. If you will need more time than this to complete your revisions, please reply to this message or contact the journal office at plosone@plos.org . When you're ready to submit your revision, log on to https://www.editorialmanager.com/pone/ and select the 'Submissions Needing Revision' folder to locate your manuscript file.

Please include the following items when submitting your revised manuscript:

- A rebuttal letter that responds to each point raised by the academic editor and reviewer(s). You should upload this letter as a separate file labeled 'Response to Reviewers'.
- A marked-up copy of your manuscript that highlights changes made to the original version. You should upload this as a separate file labeled 'Revised Manuscript with Track Changes'.
- An unmarked version of your revised paper without tracked changes. You should upload this as a separate file labeled 'Manuscript'.

If you would like to make changes to your financial disclosure, please include your updated statement in your cover letter. Guidelines for resubmitting your figure files are available below the reviewer comments at the end of this letter.

If applicable, we recommend that you deposit your laboratory protocols in protocols.io to enhance the reproducibility of your results. Protocols.io assigns your protocol its own identifier (DOI) so that it can be cited independently in the future. For instructions see: https://journals.plos.org/plosone/s/submission-guidelines#loc-laboratory-protocols . Additionally, PLOS ONE offers an option for publishing peer-reviewed Lab Protocol articles, which describe protocols hosted on protocols.io. Read more information on sharing protocols at https://plos.org/protocols?utm_medium=editorial-email&utm_source=authorletters&utm_campaign=protocols .

We look forward to receiving your revised manuscript.

Kind regards,

Zeki Akyildiz

Academic Editor

PLOS ONE

Journal Requirements:

When submitting your revision, we need you to address these additional requirements.

1.Please ensure that your manuscript meets PLOS ONE's style requirements, including those for file naming. The PLOS ONE style templates can be found at

https://journals.plos.org/plosone/s/file?id=wjVg/PLOSOne_formatting_sample_main_body.pdf and

https://journals.plos.org/plosone/s/file?id=ba62/PLOSOne_formatting_sample_title_authors_affiliations.pdf

2. We note that your Data Availability Statement is currently as follows: All relevant data are within the manuscript and in Supporting Information files.

Please confirm at this time whether or not your submission contains all raw data required to replicate the results of your study. Authors must share the “minimal data set” for their submission. PLOS defines the minimal data set to consist of the data required to replicate all study findings reported in the article, as well as related metadata and methods (https://journals.plos.org/plosone/s/data-availability#loc-minimal-data-set-definition).

For example, authors should submit the following data:

- The values behind the means, standard deviations and other measures reported;

- The values used to build graphs;

- The points extracted from images for analysis.

Authors do not need to submit their entire data set if only a portion of the data was used in the reported study.

If your submission does not contain these data, please either upload them as Supporting Information files or deposit them to a stable, public repository and provide us with the relevant URLs, DOIs, or accession numbers. For a list of recommended repositories, please see https://journals.plos.org/plosone/s/recommended-repositories.

If there are ethical or legal restrictions on sharing a de-identified data set, please explain them in detail (e.g., data contain potentially sensitive information, data are owned by a third-party organization, etc.) and who has imposed them (e.g., an ethics committee). Please also provide contact information for a data access committee, ethics committee, or other institutional body to which data requests may be sent. If data are owned by a third party, please indicate how others may request data access.

3. Please include captions for your Supporting Information files at the end of your manuscript, and update any in-text citations to match accordingly. Please see our Supporting Information guidelines for more information: http://journals.plos.org/plosone/s/supporting-information.

Please review your reference list to ensure that it is complete and correct. If you have cited papers that have been retracted, please include the rationale for doing so in the manuscript text, or remove these references and replace them with relevant current references. Any changes to the reference list should be mentioned in the rebuttal letter that accompanies your revised manuscript. If you need to cite a retracted article, indicate the article’s retracted status in the References list and also include a citation and full reference for the retraction notice.

Additional Editor Comments:

The reviewers have evaluated your research and indicated that it requires minor revisions. I will review it again once the corrections have been made.

[Note: HTML markup is below. Please do not edit.]

Reviewers' comments:

Reviewer's Responses to Questions

**Comments to the Author**

1. Is the manuscript technically sound, and do the data support the conclusions?

The manuscript must describe a technically sound piece of scientific research with data that supports the conclusions. Experiments must have been conducted rigorously, with appropriate controls, replication, and sample sizes. The conclusions must be drawn appropriately based on the data presented. 

Reviewer #1: Yes

Reviewer #2: Yes

**********

2. Has the statistical analysis been performed appropriately and rigorously? 

Reviewer #1: Yes

Reviewer #2: Yes

**********

3. Have the authors made all data underlying the findings in their manuscript fully available?

The PLOS Data policy requires authors to make all data underlying the findings described in their manuscript fully available without restriction, with rare exception (please refer to the Data Availability Statement in the manuscript PDF file). The data should be provided as part of the manuscript or its supporting information, or deposited to a public repository. For example, in addition to summary statistics, the data points behind means, medians and variance measures should be available. If there are restrictions on publicly sharing data—e.g. participant privacy or use of data from a third party—those must be specified.

Reviewer #1: No

Reviewer #2: Yes

**********

4. Is the manuscript presented in an intelligible fashion and written in standard English?

PLOS ONE does not copyedit accepted manuscripts, so the language in submitted articles must be clear, correct, and unambiguous. Any typographical or grammatical errors should be corrected at revision, so please note any specific errors here.

Reviewer #1: Yes

Reviewer #2: Yes

**********

5. Review Comments to the Author

Please use the space provided to explain your answers to the questions above. You may also include additional comments for the author, including concerns about dual publication, research ethics, or publication ethics. (Please upload your review as an attachment if it exceeds 20,000 characters)

Reviewer #1: The followings are my questions to the authors:

1. Could you clarify the abbreviation "PPD" used in the abstract?

2. Why was a 250 ms window chosen for analysis? What are the implications if a subject reaches peak force significantly earlier than 250 ms? Were such cases observed in the study?

3. Would a graphical representation of the three methods, incorporating CMJ and IMTP force-time curves, enhance the understanding of the methodologies?

4. Why was a 5SD threshold selected for force onset detection instead of manual detection? What are the advantages or limitations of this approach in particular to this new method in this study?

5. As noted by Bishop (2024), ratio data can present certain limitations. Does the newly proposed method address these issues more effectively than existing methods?

6. In the tables, including range statistics could provide additional insight. Could you explain the implications of obtaining a DSI value greater than 1?

7. The presented supplemental data appear to focus solely on ratios. Would it be beneficial to also include the raw data used to calculate these ratios?

8. Do the previously established 0.6 and 0.8 thresholds directly correspond to the new method, or have they been adjusted?

9. DOI number should be completed in references number 5 in the references list.

Reviewer #2: It seems that study design, evaluations and analysis were conducted in sound and profound fashion. The detail level about the conducted tests and measurements seems sufficient.

In order to increase the quality of the manuscript, you can find my recommendations below:

The Methods section should include information on the pre-test warm-up and nutrition, as both can have a substantial effect on the results.

The objective sentence (at the end of the introduction section) must be strengthened and must give more context about the importance and possible beneficial effects of the study

At the end of the conclusions section, please provide point to point recommendations to sport scientists about how to make further adjustments to properly improve the quality of potential future research on this (or similar) topic.

Best wishes

**********

6. PLOS authors have the option to publish the peer review history of their article (what does this mean? ). If published, this will include your full peer review and any attached files.

If you choose “no”, your identity will remain anonymous but your review may still be made public.

**Do you want your identity to be public for this peer review?** For information about this choice, including consent withdrawal, please see our Privacy Policy .

Reviewer #1: No

Reviewer #2: No

**********

[NOTE: If reviewer comments were submitted as an attachment file, they will be attached to this email and accessible via the submission site. Please log into your account, locate the manuscript record, and check for the action link "View Attachments". If this link does not appear, there are no attachment files.]

While revising your submission, please upload your figure files to the Preflight Analysis and Conversion Engine (PACE) digital diagnostic tool, https://pacev2.apexcovantage.com/ . PACE helps ensure that figures meet PLOS requirements. To use PACE, you must first register as a user. Registration is free. Then, login and navigate to the UPLOAD tab, where you will find detailed instructions on how to use the tool. If you encounter any issues or have any questions when using PACE, please email PLOS at figures@plos.org . Please note that Supporting Information files do not need this step.

# Author comment: Author response to Decision Letter 1

**DOI:** 10.1371/journal.pone.0331519.r002

9 May 2025

We thank both reviewer for their time and effort in reviewing our manuscript, we think it is now stronger with greater clarity in reporting and scientific rigour.

Please see replies and to all questions and comments below.

Reviewer #1: The followings are my questions to the authors:

1. Could you clarify the abbreviation "PPD" used in the abstract?

Thank you for noting this oversight, we have actually removed the abbreviation as it was only used once.

2. Why was a 250 ms window chosen for analysis? What are the implications if a subject reaches peak force significantly earlier than 250 ms? Were such cases observed in the study?

A 250 ms window was selected as a threshold as this is the commonly recognised threshold for fast stretch shortening cycle actions (i.e. plyometric actions). We have clarified this within the text with supporting reference.

Although this is an interesting point, it is highly unlikely that peak force will occur < 250 ms. No participants within the present study displayed this characteristic, this is consistent with previous literature in the isometric midthigh pull (Comfort et al., 2019) and in single joint actions (Andersen & Aagaard, 2006).

Comfort, P., Dos’Santos, T., Jones, P. A., McMahon, J. J., Suchomel, T. J., Bazyler, C., & Stone, M. H. (2020). Normalization of Early Isometric Force Production as a Percentage of Peak Force During Multijoint Isometric Assessment. International Journal of Sports Physiology and Performance, 15(4), 478-482.

Andersen LL, Aagaard P (2006) Influence of maximal muscle strength and intrinsic muscle contractile properties on contractile rate of force development. Eur J Appl Physiol 96:46–52

3. Would a graphical representation of the three methods, incorporating CMJ and IMTP force-time curves, enhance the understanding of the methodologies?

Although this could be an interesting addition, we do not think it is necceasry as it has been previously illustrated effectively in the the work from James and Comfort (2022)

James LP, Comfort P. The reliability of novel, temporal-based dynamic strength index metrics. Sports Biomechanics. 2022;4(Feb):1-12.

4. Why was a 5SD threshold selected for force onset detection instead of manual detection? What are the advantages or limitations of this approach in particular to this new method in this study?

This threshold was used as it is recommended by Owen et al. (2014), which is a standard protocol for CMJ assessment.

5. As noted by Bishop (2024), ratio data can present certain limitations. Does the newly proposed method address these issues more effectively than existing methods?

Despite issues with ratio data it can still be useful and it is recommended that practitioners consider the constituent parts of DSI, and we acknowledge the potential that iDSI might be making it more complex (lines 423-430). We have included the Bishop reference to support this point.

“Further, a limitation of ratio-based metrics, such as the DSI, is that the practitioner needs to consider the constituent parts with an fDSI. In this scenario, the ability to interpret peak force (either absolute or relative) is more simplistic than an iDSI variation. Hence, the iDSI could be making interpretation of an athlete isometric and ballistic force generating capabilities more complex for practitioners. Similarly, observing the absolute performance of the dynamic task, such as the CMJ, would also be imperative with both fDSI and iDSI variations, as jump height, time to take off and propulsive phase duration highlight an athlete’s wider performance characteristics.”

6. In the tables, including range statistics could provide additional insight. Could you explain the implications of obtaining a DSI value greater than 1?

We have now included the range statistics. A comment around the achieving a DSI >1 has now been included within the introduction lines 91-93.

“If a DSI score is greater than 1.00, this could be representative of either lack of understanding and familiarization within the isometric task or adopting an overly stiff strategy in the ballistic task, as isometric forces should be greater than dynamic force production [5].”

7. The presented supplemental data appear to focus solely on ratios. Would it be beneficial to also include the raw data used to calculate these ratios?

We have now included the data as well.

8. Do the previously established 0.6 and 0.8 thresholds directly correspond to the new method, or have they been adjusted?

They have not been adjusted as there is no current recommendations to adjust, even when using different protocols. These are the recommendations from Comfort et al. (2018)

9. DOI number should be completed in references number 5 in the references list.

This has now been updated.

Reviewer #2: It seems that study design, evaluations and analysis were conducted in sound and profound fashion. The detail level about the conducted tests and measurements seems sufficient.

In order to increase the quality of the manuscript, you can find my recommendations below:

The Methods section should include information on the pre-test warm-up and nutrition, as both can have a substantial effect on the results.

We have provided some brief information on nutrition, however, as this is performed within-session (1 session) and not between nutrition would have a minimal effect on performance. We have provided detail on the warm.

The objective sentence (at the end of the introduction section) must be strengthened and must give more context about the importance and possible beneficial effects of the study

A practical beneficial statement has been added to this section.

At the end of the conclusions section, please provide point to point recommendations to sport scientists about how to make further adjustments to properly improve the quality of potential future research on this (or similar) topic.

Future research has been identified at the end of the conclusion. With appropriate suggestions on further adjustments and requirements to observe the constituent parts. Lines 458-466.

“Whereas iDSI matched could result in similar DSI values even following training as the ratio will compare force generating characteristics over identical durations, hence the need to observe the constituent parts of the ratio metric. However, currently both iDSI fixed and iDSI matched need to further investigation to determine if the same absolute thresholds for determining training recommendations can be used for iDSI. Moreover, future research should also be conducted to determine the efficacy of training guidelines when using iDSI to determine if the greater context provided to an athlete’s dynamic force generating capability results in greater training effectiveness.”

Best wishes

Attachment Submitted filename: Response to reviewers.docx

# Peer review: Decision Letter 1

**DOI:** 10.1371/journal.pone.0331519.r003

**Authors:** Zeki Akyildiz

29 May 2025

PONE-D-25-07136R1Comparisons between different methods of calculating dynamic strength index: Effect on training recommendationsPLOS ONE

Dear Dr. Ripley,

Thank you for submitting your manuscript to PLOS ONE. After careful consideration, we feel that it has merit but does not fully meet PLOS ONE’s publication criteria as it currently stands. Therefore, we invite you to submit a revised version of the manuscript that addresses the points raised during the review process.

Please submit your revised manuscript by Jul 13 2025 11:59PM. If you will need more time than this to complete your revisions, please reply to this message or contact the journal office at plosone@plos.org . When you're ready to submit your revision, log on to https://www.editorialmanager.com/pone/ and select the 'Submissions Needing Revision' folder to locate your manuscript file.

Please include the following items when submitting your revised manuscript:

- A rebuttal letter that responds to each point raised by the academic editor and reviewer(s). You should upload this letter as a separate file labeled 'Response to Reviewers'.
- A marked-up copy of your manuscript that highlights changes made to the original version. You should upload this as a separate file labeled 'Revised Manuscript with Track Changes'.
- An unmarked version of your revised paper without tracked changes. You should upload this as a separate file labeled 'Manuscript'.

If you would like to make changes to your financial disclosure, please include your updated statement in your cover letter. Guidelines for resubmitting your figure files are available below the reviewer comments at the end of this letter.

If applicable, we recommend that you deposit your laboratory protocols in protocols.io to enhance the reproducibility of your results. Protocols.io assigns your protocol its own identifier (DOI) so that it can be cited independently in the future. For instructions see: https://journals.plos.org/plosone/s/submission-guidelines#loc-laboratory-protocols . Additionally, PLOS ONE offers an option for publishing peer-reviewed Lab Protocol articles, which describe protocols hosted on protocols.io. Read more information on sharing protocols at https://plos.org/protocols?utm_medium=editorial-email&utm_source=authorletters&utm_campaign=protocols .

We look forward to receiving your revised manuscript.

Kind regards,

Zeki Akyildiz

Academic Editor

PLOS ONE

Journal Requirements:

Please review your reference list to ensure that it is complete and correct. If you have cited papers that have been retracted, please include the rationale for doing so in the manuscript text, or remove these references and replace them with relevant current references. Any changes to the reference list should be mentioned in the rebuttal letter that accompanies your revised manuscript. If you need to cite a retracted article, indicate the article’s retracted status in the References list and also include a citation and full reference for the retraction notice.

Additional Editor Comments:

Please address the points made by the reviewers I agree with the reviewers request for graphics. I recommend that you make any necessary revisions to ensure that readers can best understand the article.

[Note: HTML markup is below. Please do not edit.]

Reviewers' comments:

Reviewer's Responses to Questions

**Comments to the Author**

1. If the authors have adequately addressed your comments raised in a previous round of review and you feel that this manuscript is now acceptable for publication, you may indicate that here to bypass the “Comments to the Author” section, enter your conflict of interest statement in the “Confidential to Editor” section, and submit your "Accept" recommendation.

Reviewer #1: All comments have been addressed

Reviewer #2: All comments have been addressed

**********

2. Is the manuscript technically sound, and do the data support the conclusions?

The manuscript must describe a technically sound piece of scientific research with data that supports the conclusions. Experiments must have been conducted rigorously, with appropriate controls, replication, and sample sizes. The conclusions must be drawn appropriately based on the data presented. 

Reviewer #1: Yes

Reviewer #2: Yes

**********

3. Has the statistical analysis been performed appropriately and rigorously? 

Reviewer #1: Yes

Reviewer #2: Yes

**********

4. Have the authors made all data underlying the findings in their manuscript fully available?

The PLOS Data policy requires authors to make all data underlying the findings described in their manuscript fully available without restriction, with rare exception (please refer to the Data Availability Statement in the manuscript PDF file). The data should be provided as part of the manuscript or its supporting information, or deposited to a public repository. For example, in addition to summary statistics, the data points behind means, medians and variance measures should be available. If there are restrictions on publicly sharing data—e.g. participant privacy or use of data from a third party—those must be specified.

Reviewer #1: Yes

Reviewer #2: Yes

**********

5. Is the manuscript presented in an intelligible fashion and written in standard English?

PLOS ONE does not copyedit accepted manuscripts, so the language in submitted articles must be clear, correct, and unambiguous. Any typographical or grammatical errors should be corrected at revision, so please note any specific errors here.

Reviewer #1: Yes

Reviewer #2: Yes

**********

6. Review Comments to the Author

Please use the space provided to explain your answers to the questions above. You may also include additional comments for the author, including concerns about dual publication, research ethics, or publication ethics. (Please upload your review as an attachment if it exceeds 20,000 characters)

Reviewer #1: (No Response)

Reviewer #2: I recommend it to be accepted in its current form. Thank you for revisions. I believe that this manuscript can be a valuable addition to the practical sport science applications related to understanding the role different methods of calculating dynamic strength index.

Best wishes.

**********

7. PLOS authors have the option to publish the peer review history of their article (what does this mean? ). If published, this will include your full peer review and any attached files.

If you choose “no”, your identity will remain anonymous but your review may still be made public.

**Do you want your identity to be public for this peer review?** For information about this choice, including consent withdrawal, please see our Privacy Policy .

Reviewer #1: No

Reviewer #2: No

**********

[NOTE: If reviewer comments were submitted as an attachment file, they will be attached to this email and accessible via the submission site. Please log into your account, locate the manuscript record, and check for the action link "View Attachments". If this link does not appear, there are no attachment files.]

While revising your submission, please upload your figure files to the Preflight Analysis and Conversion Engine (PACE) digital diagnostic tool, https://pacev2.apexcovantage.com/ . PACE helps ensure that figures meet PLOS requirements. To use PACE, you must first register as a user. Registration is free. Then, login and navigate to the UPLOAD tab, where you will find detailed instructions on how to use the tool. If you encounter any issues or have any questions when using PACE, please email PLOS at figures@plos.org . Please note that Supporting Information files do not need this step.

# Author comment: Author response to Decision Letter 2

**DOI:** 10.1371/journal.pone.0331519.r004

30 May 2025

We thank both reviewers for accepting the manuscript in its current form highlighting no further amendments were required. At the editors request we have improved the graphics of the included figures which both reviewers have accepted and hope this is sufficient.

Thank you.

Attachment Submitted filename: Response to editor and reviewer.docx

# Peer review: Decision Letter 2

**DOI:** 10.1371/journal.pone.0331519.r005

**Authors:** Javier Peña

23 Jul 2025

PONE-D-25-07136R2Comparisons between different methods of calculating dynamic strength index: Effect on training recommendationsPLOS ONE

Dear Dr. Ripley,

Thank you for submitting your manuscript to PLOS ONE. After careful consideration, we feel that it has merit but does not fully meet PLOS ONE’s publication criteria as it currently stands. Specifically, comments from Reviewer 1 were not available for the authors, due to some errors in the use of the editorial manager. Please, read carefully and address the comments I paste below for your convenience.

==============================

REVIEWER 1: Unfortunately, I could not thoroughly review the second revision of the manuscript because the authors did not receive my comments.

I provided the comments below (at the end) on the first revision of the manuscript. As I selected "Accept," I included my comments in the "Confidential to Editor" section, intending for them to be filtered by the Editor.

In the decision letter, the Editor referenced my comments, stating, “Please address the points made by the reviewers. I agree with the reviewers' request for graphics. I recommend that you make any necessary revisions to ensure that readers can best understand the article.”

However, it appears that the authors did not receive my comments. Consequently, the authors did not include a new graph in the second version of the manuscript.

My comments to the first revision of the manuscript:

I raised this point for the first version: “Would a graphical representation of the three methods, incorporating CMJ and IMTP force-time curves, enhance the understanding of the methodologies?”

The authors' response was: “Although this could be an interesting addition, we do not think it is necceasry as it has been previously illustrated effectively in the the work from James and Comfort (2022) James LP, Comfort P. The reliability of novel, temporal-based dynamic strength index metrics. Sports Biomechanics. 2022;4(Feb):1-12”

I still think a graphical representation would be useful. Unlike PLOS One, the article in which it is mentioned is not open source and is therefore not available to anyone. I understand that creating such a graph would be time-consuming, and the authors would prefer to publish sooner rather than later. In the long term, though, a graph would benefit authors and readers. Ultimately, the editors can make the final decision.

I raised another point for the first version: “Why was a 5SD threshold selected for force onset detection instead of manual detection? What are the advantages or limitations of this approach in particular to this new method in this study?”

The authors' response was: “This threshold was used as it is recommended by Owen et al. (2014), which is a standard protocol for CMJ assessment.” 

I did not refer to the CMJ analysis. I referred to the IMTP analysis (For the IMTP, the onset of force production was defined as an increase in force that was greater than five standard deviations (SD) of the mean force calculated during the last 1 second

immediately before the pull commence [20]). Using 5 SD is not as standard as it is IMTP, unlike CMJ. Due to fluctuations in force caused by pre-tension and other factors, manual approaches are also common for force onset detection in IMTP analysis.

==============================

Please submit your revised manuscript by Sep 06 2025 11:59PM. If you will need more time than this to complete your revisions, please reply to this message or contact the journal office at plosone@plos.org . When you're ready to submit your revision, log on to https://www.editorialmanager.com/pone/ and select the 'Submissions Needing Revision' folder to locate your manuscript file.

Please include the following items when submitting your revised manuscript:

- A rebuttal letter that responds to each point raised by the academic editor and reviewer(s). You should upload this letter as a separate file labeled 'Response to Reviewers'.
- A marked-up copy of your manuscript that highlights changes made to the original version. You should upload this as a separate file labeled 'Revised Manuscript with Track Changes'.
- An unmarked version of your revised paper without tracked changes. You should upload this as a separate file labeled 'Manuscript'.

If you would like to make changes to your financial disclosure, please include your updated statement in your cover letter. Guidelines for resubmitting your figure files are available below the reviewer comments at the end of this letter.

If applicable, we recommend that you deposit your laboratory protocols in protocols.io to enhance the reproducibility of your results. Protocols.io assigns your protocol its own identifier (DOI) so that it can be cited independently in the future. For instructions see: https://journals.plos.org/plosone/s/submission-guidelines#loc-laboratory-protocols . Additionally, PLOS ONE offers an option for publishing peer-reviewed Lab Protocol articles, which describe protocols hosted on protocols.io. Read more information on sharing protocols at https://plos.org/protocols?utm_medium=editorial-email&utm_source=authorletters&utm_campaign=protocols .

We look forward to receiving your revised manuscript.

Kind regards,

Javier Peña, Ph.D.

Academic Editor

PLOS ONE

**Journal Requirements:**

1. If the reviewer comments include a recommendation to cite specific previously published works, please review and evaluate these publications to determine whether they are relevant and should be cited. There is no requirement to cite these works unless the editor has indicated otherwise. 

2. Please review your reference list to ensure that it is complete and correct. If you have cited papers that have been retracted, please include the rationale for doing so in the manuscript text, or remove these references and replace them with relevant current references. Any changes to the reference list should be mentioned in the rebuttal letter that accompanies your revised manuscript. If you need to cite a retracted article, indicate the article’s retracted status in the References list and also include a citation and full reference for the retraction notice.

[Note: HTML markup is below. Please do not edit.]

Reviewers' comments:

Reviewer's Responses to Questions

**Comments to the Author**

1. If the authors have adequately addressed your comments raised in a previous round of review and you feel that this manuscript is now acceptable for publication, you may indicate that here to bypass the “Comments to the Author” section, enter your conflict of interest statement in the “Confidential to Editor” section, and submit your "Accept" recommendation.

Reviewer #1: All comments have been addressed

Reviewer #2: All comments have been addressed

**********

2. Is the manuscript technically sound, and do the data support the conclusions?

The manuscript must describe a technically sound piece of scientific research with data that supports the conclusions. Experiments must have been conducted rigorously, with appropriate controls, replication, and sample sizes. The conclusions must be drawn appropriately based on the data presented. 

Reviewer #1: Yes

Reviewer #2: Yes

**********

3. Has the statistical analysis been performed appropriately and rigorously? 

Reviewer #1: Yes

Reviewer #2: Yes

**********

4. Have the authors made all data underlying the findings in their manuscript fully available?

The PLOS Data policy requires authors to make all data underlying the findings described in their manuscript fully available without restriction, with rare exception (please refer to the Data Availability Statement in the manuscript PDF file). The data should be provided as part of the manuscript or its supporting information, or deposited to a public repository. For example, in addition to summary statistics, the data points behind means, medians and variance measures should be available. If there are restrictions on publicly sharing data—e.g. participant privacy or use of data from a third party—those must be specified.

Reviewer #1: Yes

Reviewer #2: Yes

**********

5. Is the manuscript presented in an intelligible fashion and written in standard English?

PLOS ONE does not copyedit accepted manuscripts, so the language in submitted articles must be clear, correct, and unambiguous. Any typographical or grammatical errors should be corrected at revision, so please note any specific errors here.

Reviewer #1: Yes

Reviewer #2: Yes

**********

6. Review Comments to the Author

Please use the space provided to explain your answers to the questions above. You may also include additional comments for the author, including concerns about dual publication, research ethics, or publication ethics. (Please upload your review as an attachment if it exceeds 20,000 characters)

**Reviewer #1: ** Unfortunately, I could not thoroughly review the second revision of the manuscript because the authors did not receive my comments.

I provided the comments below (at the end) on the first revision of the manuscript. As I selected "Accept," I included my comments in the "Confidential to Editor" section, intending for them to be filtered by the Editor.

In the decision letter, the Editor referenced my comments, stating, “Please address the points made by the reviewers. I agree with the reviewers' request for graphics. I recommend that you make any necessary revisions to ensure that readers can best understand the article.”

However, it appears that the authors did not receive my comments. Consequently, the authors did not include a new graph in the second version of the manuscript.

My comments to the first revision of the manuscript:

I raised this point for the first version: “Would a graphical representation of the three methods, incorporating CMJ and IMTP force-time curves, enhance the understanding of the methodologies?”

The authors' response was: “Although this could be an interesting addition, we do not think it is necceasry as it has been previously illustrated effectively in the the work from James and Comfort (2022) James LP, Comfort P. The reliability of novel, temporal-based dynamic strength index metrics. Sports Biomechanics. 2022;4(Feb):1-12”

I still think a graphical representation would be useful. Unlike PLOS One, the article in which it is mentioned is not open source and is therefore not available to anyone. I understand that creating such a graph would be time-consuming, and the authors would prefer to publish sooner rather than later. In the long term, though, a graph would benefit authors and readers. Ultimately, the editors can make the final decision.

I raised another point for the first version: “Why was a 5SD threshold selected for force onset detection instead of manual detection? What are the advantages or limitations of this approach in particular to this new method in this study?”

The authors' response was: “This threshold was used as it is recommended by Owen et al. (2014), which is a standard protocol for CMJ assessment.”

I did not refer to the CMJ analysis. I referred to the IMTP analysis (For the IMTP, the onset of force production was defined as an increase in force that was greater than five standard deviations (SD) of the mean force calculated during the last 1 second

immediately before the pull commence [20]). Using 5 SD is not as standard as it is IMTP, unlike CMJ. Due to fluctuations in force caused by pre-tension and other factors, manual approaches are also common for force onset detection in IMTP analysis.

**Reviewer #2: ** Dear Authors

It seems that the suggested revisions were made successfully. With its current form, I believe that this manuscript can be a valuable addition to the practical sport science applications.

Best wishes.

**********

7. PLOS authors have the option to publish the peer review history of their article (what does this mean? ). If published, this will include your full peer review and any attached files.

If you choose “no”, your identity will remain anonymous but your review may still be made public.

**Do you want your identity to be public for this peer review?** For information about this choice, including consent withdrawal, please see our Privacy Policy .

Reviewer #1: No

Reviewer #2: No

**********

[NOTE: If reviewer comments were submitted as an attachment file, they will be attached to this email and accessible via the submission site. Please log into your account, locate the manuscript record, and check for the action link "View Attachments". If this link does not appear, there are no attachment files.]

While revising your submission, please upload your figure files to the Preflight Analysis and Conversion Engine (PACE) digital diagnostic tool, https://pacev2.apexcovantage.com/ . PACE helps ensure that figures meet PLOS requirements. To use PACE, you must first register as a user. Registration is free. Then, login and navigate to the UPLOAD tab, where you will find detailed instructions on how to use the tool. If you encounter any issues or have any questions when using PACE, please email PLOS at figures@plos.org . Please note that Supporting Information files do not need this step.

# Author comment: Author response to Decision Letter 3

**DOI:** 10.1371/journal.pone.0331519.r006

1 Aug 2025

Dear reviewer,

We have provided a detailed response to the feedback included below, obviously we would have done this in the first review if we had received all these comments with enough detail.

We thank the reviewer for the discourse and comments highlighted.

REVIEWER 1:

Unfortunately, I could not thoroughly review the second revision of the manuscript because the authors did not receive my comments.

I provided the comments below (at the end) on the first revision of the manuscript. As I selected "Accept," I included my comments in the "Confidential to Editor" section, intending for them to be filtered by the Editor.

In the decision letter, the Editor referenced my comments, stating, “Please address the points made by the reviewers. I agree with the reviewers' request for graphics. I recommend that you make any necessary revisions to ensure that readers can best understand the article.”

However, it appears that the authors did not receive my comments. Consequently, the authors did not include a new graph in the second version of the manuscript.

Response - Although we acknowledge this oversight and it unfortunate that we did not receive this on the first review, we do want to make the reviewer aware that the handling editor has changed multiple times since submission and to this point (three different editors).

My comments to the first revision of the manuscript:

I raised this point for the first version: “Would a graphical representation of the three methods, incorporating CMJ and IMTP force-time curves, enhance the understanding of the methodologies?”

The authors' response was: “Although this could be an interesting addition, we do not think it is necceasry as it has been previously illustrated effectively in the the work from James and Comfort (2022) James LP, Comfort P. The reliability of novel, temporal-based dynamic strength index metrics. Sports Biomechanics. 2022;4(Feb):1-12”

I still think a graphical representation would be useful. Unlike PLOS One, the article in which it is mentioned is not open source and is therefore not available to anyone. I understand that creating such a graph would be time-consuming, and the authors would prefer to publish sooner rather than later. In the long term, though, a graph would benefit authors and readers. Ultimately, the editors can make the final decision.

Response - Although we do agree with our previous response around their being previously published figures. However, as the reviewer does feel strongly around this, we have created our figures which replicate those published previously. We do want to highlight that although the article is not open access, it is available on request from the authors, and it is not the responsibility of authors of newer works to replicate work that is behind a “pay wall” from other publishers.

I raised another point for the first version: “Why was a 5SD threshold selected for force onset detection instead of manual detection? What are the advantages or limitations of this approach in particular to this new method in this study?”

The authors' response was: “This threshold was used as it is recommended by Owen et al. (2014), which is a standard protocol for CMJ assessment.”

I did not refer to the CMJ analysis. I referred to the IMTP analysis (For the IMTP, the onset of force production was defined as an increase in force that was greater than five standard deviations (SD) of the mean force calculated during the last 1 second

immediately before the pull commence [20]). Using 5 SD is not as standard as it is IMTP, unlike CMJ. Due to fluctuations in force caused by pre-tension and other factors, manual approaches are also common for force onset detection in IMTP analysis.

Response - We apologies for the confusion based on the response, but as we used thresholds for both the CMJ and IMTP and your comment (which was provided by the editor) did not specify if it was the CMJ or IMTP we made a best guess and we overlooked the threshold for the IMTP when responding or we would have made this clearer.

Although the reviewer highlights some of the issues with a calculated onset threshold, you incorrectly suggest there no standard protocols for this, when in fact identifying onset through 5SD are the standardized methods recommended by Comfort et al. (2019). A threshold of 5 SD has also been supported in the literature by the work of Dos’Santos et al. (2017), suggesting to use this threshold over manual identification. Interestingly, Guppy et al. (2018) found both 5SD and 3SD both agree with manual identification for time at force onset, while automated threshold also had improved reliability in force at longer epochs (i.e., >200 ms). Therefore, 5SD was used for the present study. We have now included both the Dos’Santos et al. (2017) and Guppy et al. (2018) references to support theuse of the 5SD onset threshold.

Comfort P, Dos’Santos T, Beckham GK, Stone M, Guppy S, Haff G. Standardization and Methodological Considerations for the Isometric Midthigh Pull. Strength and Conditioning Journal. 2019;41(2):1.

DosʼSantos T, Jones PA, Comfort P, Thomas C. Effect of Different Onset Thresholds on Isometric Midthigh Pull Force-Time Variables. J Strength Cond Res. 2017 Dec;31(12):3463-3473. doi: 10.1519/JSC.0000000000001765.

Guppy, S. N., Brady, C. J., Kotani, Y., Connolly, S., Comfort, P., Lake, J. P., & Haff, G. G. (2024). A comparison of manual and automatic force-onset identification methodologies and their effect on force-time characteristics in the isometric midthigh pull. Sports Biomechanics, 23(10), 1663-1680. https://doi.org/10.1080/14763141.2021.1974532

Attachment Submitted filename: Response R3.docx

# Peer review: Decision Letter 3

**DOI:** 10.1371/journal.pone.0331519.r007

**Authors:** Emiliano Cè

17 Aug 2025

Comparisons between different methods of calculating dynamic strength index: Effect on training recommendations

PONE-D-25-07136R3

Dear Dr. Ripley,

We’re pleased to inform you that your manuscript has been judged scientifically suitable for publication and will be formally accepted for publication once it meets all outstanding technical requirements.

Within one week, you’ll receive an e-mail detailing the required amendments. When these have been addressed, you’ll receive a formal acceptance letter and your manuscript will be scheduled for publication.

An invoice will be generated when your article is formally accepted. Please note, if your institution has a publishing partnership with PLOS and your article meets the relevant criteria, all or part of your publication costs will be covered. Please make sure your user information is up-to-date by logging into Editorial Manager at Editorial Manager®  and clicking the ‘Update My Information' link at the top of the page. For questions related to billing, please contact billing support .

If your institution or institutions have a press office, please notify them about your upcoming paper to help maximize its impact. If they’ll be preparing press materials, please inform our press team as soon as possible -- no later than 48 hours after receiving the formal acceptance. Your manuscript will remain under strict press embargo until 2 pm Eastern Time on the date of publication. For more information, please contact onepress@plos.org.

Kind regards,

Emiliano Cè, Ph.D.

Academic Editor

PLOS ONE

Additional Editor Comments (optional):

Reviewers' comments:

Reviewer's Responses to Questions

**Comments to the Author**

1. If the authors have adequately addressed your comments raised in a previous round of review and you feel that this manuscript is now acceptable for publication, you may indicate that here to bypass the “Comments to the Author” section, enter your conflict of interest statement in the “Confidential to Editor” section, and submit your "Accept" recommendation.

Reviewer #1: All comments have been addressed

Reviewer #2: All comments have been addressed

**********

2. Is the manuscript technically sound, and do the data support the conclusions?

The manuscript must describe a technically sound piece of scientific research with data that supports the conclusions. Experiments must have been conducted rigorously, with appropriate controls, replication, and sample sizes. The conclusions must be drawn appropriately based on the data presented. 

Reviewer #1: Yes

Reviewer #2: Yes

**********

3. Has the statistical analysis been performed appropriately and rigorously? 

Reviewer #1: Yes

Reviewer #2: Yes

**********

4. Have the authors made all data underlying the findings in their manuscript fully available?

The PLOS Data policy requires authors to make all data underlying the findings described in their manuscript fully available without restriction, with rare exception (please refer to the Data Availability Statement in the manuscript PDF file). The data should be provided as part of the manuscript or its supporting information, or deposited to a public repository. For example, in addition to summary statistics, the data points behind means, medians and variance measures should be available. If there are restrictions on publicly sharing data—e.g. participant privacy or use of data from a third party—those must be specified.

Reviewer #1: Yes

Reviewer #2: Yes

**********

5. Is the manuscript presented in an intelligible fashion and written in standard English?

PLOS ONE does not copyedit accepted manuscripts, so the language in submitted articles must be clear, correct, and unambiguous. Any typographical or grammatical errors should be corrected at revision, so please note any specific errors here.

Reviewer #1: Yes

Reviewer #2: Yes

**********

6. Review Comments to the Author

Please use the space provided to explain your answers to the questions above. You may also include additional comments for the author, including concerns about dual publication, research ethics, or publication ethics. (Please upload your review as an attachment if it exceeds 20,000 characters)

Reviewer #1: The Manuscript can be accepted in its current form. The authors addressed all the issues raised by the reviewers.

Reviewer #2: Dear Authors,

It appears that the proposed changes were effectively implemented. In its present state, I am confident that this manuscript will significantly contribute to practical applications in sport science concerning dynamic strength index calculations.

Kind regards,

**********

7. PLOS authors have the option to publish the peer review history of their article (what does this mean? ). If published, this will include your full peer review and any attached files.

If you choose “no”, your identity will remain anonymous but your review may still be made public.

**Do you want your identity to be public for this peer review?** For information about this choice, including consent withdrawal, please see our Privacy Policy .

Reviewer #1: No

Reviewer #2: No

**********

# Editor report: Acceptance letter

**DOI:** 10.1371/journal.pone.0331519.r008

**Authors:** Emiliano Cè

PONE-D-25-07136R3

PLOS ONE

Dear Dr. Ripley,

I'm pleased to inform you that your manuscript has been deemed suitable for publication in PLOS ONE. Congratulations! Your manuscript is now being handed over to our production team.

At this stage, our production department will prepare your paper for publication. This includes ensuring the following:

* All references, tables, and figures are properly cited

* All relevant supporting information is included in the manuscript submission,

* There are no issues that prevent the paper from being properly typeset

You will receive further instructions from the production team, including instructions on how to review your proof when it is ready. Please keep in mind that we are working through a large volume of accepted articles, so please give us a few days to review your paper and let you know the next and final steps.

Lastly, if your institution or institutions have a press office, please let them know about your upcoming paper now to help maximize its impact. If they'll be preparing press materials, please inform our press team within the next 48 hours. Your manuscript will remain under strict press embargo until 2 pm Eastern Time on the date of publication. For more information, please contact onepress@plos.org.

You will receive an invoice from PLOS for your publication fee after your manuscript has reached the completed accept phase. If you receive an email requesting payment before acceptance or for any other service, this may be a phishing scheme. Learn how to identify phishing emails and protect your accounts at https://explore.plos.org/phishing.

If we can help with anything else, please email us at customercare@plos.org.

Thank you for submitting your work to PLOS ONE and supporting open access.

Kind regards,

PLOS ONE Editorial Office Staff

on behalf of

Prof. Emiliano Cè

Academic Editor

PLOS ONE
